# A textile-based alignment-free electrophysiological sensing sleeve for comprehensive cardiovascular monitoring

**DOI:** 10.1038/s41378-025-01088-x

**Published:** 2025-11-26

**Authors:** Shirong Qiu, Yihao Li, Chengkai Dai, Shun Wu, Xiangjia Chen, Nan Ji, Guoxin Fang, Yeung Yam, Charlie C. L. Wang, Ni Zhao

**Affiliations:** 1 Department of Electronic Engineering, The Chinese University of Hong Kong, Hong Kong SAR, China; 2 Centre for Perceptual and Interactive Intelligence (CPII) Limited, Hong Kong SAR, China; 3 Department of Mechanical and Automation Engineering, The Chinese University of Hong Kong, Hong Kong SAR, China; 4 United Sensing and MediTech Limited, Hong Kong SAR, China; 5 Department of Mechanical and Aerospace Engineering, The University of Manchester, Manchester, UK

**Keywords:** Electrical and electronic engineering, Electronic properties and materials

## Abstract

Continuous monitoring of cardiovascular risk factors in daily life is crucial for disease prevention and management. Current wearable systems, such as photoplethysmography (PPG), ultrasound, and pressure sensors, can capture some of these parameters but require precise sensor alignment over arteries. This alignment dependency complicates daily use and makes the signals highly susceptible to motion artifacts. In this work, we present a textile-based alignment-free electrophysiological sensing sleeve (TAESS) that can be comfortably worn on the upper arm. The TAESS integrates impedance plethysmography (IPG) and electrocardiography (ECG) to enable synchronized cardiovascular haemodynamic monitoring, including blood pressure (BP), cardiac output (CO), systemic vascular resistance (SVR), heart rate (HR), and other metrics. The sleeve is fabricated using silver-based conductive yarns, forming flexible, breathable, and stretchable electrodes that are produced via an automated, low-cost knitting process. Compared to commercial electrodes, TAESS demonstrates superior permeability (37.5 mg·cm^−2^·h^−1^), stretchability (exceeding 45% in wale direction), and thermal regulation (remaining within 0.4 °C after exercise). Most importantly, it maintains high signal fidelity and is minimally affected by radial movements, outperforming commercial PPG sensors in blood volume detection. The TAESS achieved systolic and diastolic BP prediction root-mean-squared errors of 7.05 mmHg and 5.93 mmHg, respectively, even under respiratory interference and after re-wearing. This scalable, low-cost sensing sleeve offers a robust and alignment-free solution for continuous cardiovascular monitoring, paving the way for personalized healthcare in daily life.

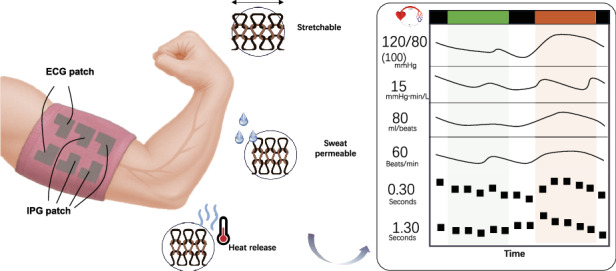

## Introduction

Cardiovascular diseases (CVDs) remain the foremost global health challenge, claiming over 17.9 million lives annually—a toll projected to reach 23.6 million by 2030^1^. This growing crisis demands innovative health monitoring technologies capable of synchronously capturing multiple physiological parameters in real-world contexts^2,3^. In clinical settings, such as intensive care units^4^, simultaneous tracking of heart rate (HR), stroke volume (SV), systemic vascular resistance (SVR), and blood pressure (BP) informs critical interventions, while in home environments^5^, these metrics enable effective management of chronic conditions like heart failure. Yet, existing wearable devices, often limited to photoplethysmography (PPG) or single-modality sensors, fall short of delivering the integrated data required for comprehensive cardiovascular assessment, particularly in dynamic or complex scenarios^6–8^.

The human body continuously emits electrophysiological signals from cardiac, neural, and muscular activities^9^, providing a rich source of physiological insight. Techniques such as electrocardiography (ECG)^10^, impedance plethysmography (IPG)^11^, electroencephalography (EEG)^12^, and electromyography (EMG) capture these signals^13^, enabling detection of arrhythmias, vascular dynamics, and neuro-muscular states. Yet, the potential of these multi-modal electrical signals remains largely untapped, as existing physiological models are limited to basic single-parameter extraction and analysis^14^. In other words, for the complex parameters such as SVR (systemic vascular resistance, a determinant of BP that is negatively correlated with blood flow and cardiac output (CO)) or SV have not been fully explored by wearable devices^4,15^. These constraints render existing sensing technologies unsuitable and unreliable for regular cardiovascular metrics measurement. Elucidating the interplay between cardiac electrical activity and blood flow dynamics^16^, for instance, could redefine diagnostics and personalized care—an ambition contingent on stable, integrated sensing platforms that current technologies fail to provide.

In this study we developed a textile-based alignment-free electrophysiological sensing sleeve (TAESS) that integrates ECG and IPG electrodes made from conductive yarns for comprehensive multi-parameter cardiovascular monitoring. While textile-based electrodes have demonstrated advances in flexibility and wearability^17,18^, their applications have largely been limited to material science improvements^19,20^ with minimal functional expansion. Nevertheless, textile-based electronics have garnered significant attention due to their unique advantages^21–24^, such as pervasive health management^22^, therapy^23^, and human-machine interface^21^. Leveraging these benefits, we designed a wearable sleeve optimized for upper-arm placement, enabling synchronous multi-parameter monitoring of HR, SV, SVR, and BP through flexible, breathable electrodes seamlessly integrated into daily wear. By leveraging the interplay between dual-modal signals—ECG for cardiac electrical activity and IPG for blood volume dynamics—our system provides synergistic physiological insights. Additionally, the automated knitted smart textile ensures low-cost manufacturing, superior signal fidelity, stretchability, and motion compatibility compared to commercial systems, establishing a scalable solution for continuous, alignment-free cardiovascular monitoring in everyday healthcare settings.

## Results

### Working principle

**Fig. 1:**
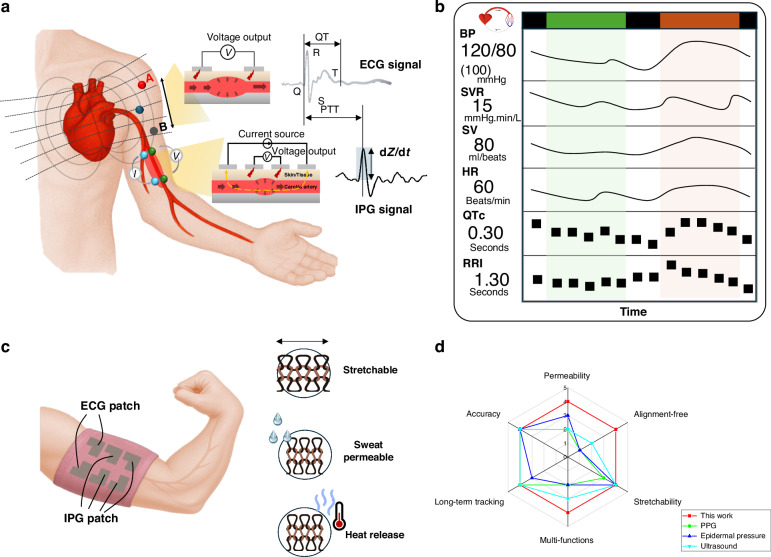
Working principle of textile-based cardiovascular heamodynamic measurement. **a** Schematic illustration of ECG and IPG signal generation in the arm and simplified signal model for cardiovascular metrics measurement. **b** Typical cardiovascular metrics measured by the TAESS system. **c** Process and layout of TAESS system. **d** Technical comparison between our system and published works utilizing photoplethysmography (PPG), ultrasound wall tracking and epidermal pressure sensor for continuous cardiovascular metrics (e.g., BP monitoring) in terms of multi-functions, accuracy, long-term track, alignment-free, permeability and stretchability

The TAESS system integrates synchronized electrocardiography (ECG) and impedance plethysmography (IPG) to enable cardiovascular monitoring, as illustrated in Fig. 1. The ECG module, designed for limb attachment via flexible fabric electrodes woven with silver-based conductive yarns, detects minute potential differences induced by cardiac myocyte depolarization. These potential differences, transduced to the body surface, are regarded as an electric dipole field^25^ and measured on the upper arm (Fig. 1a). This placement reduces electrode displacement compared to wrist-based leads, as the upper arm offers a more stable anatomical site with closer proximity to the cardiac vector projection^26^, see Fig. S1. In addition, unlike conventional watch-type single-lead ECG devices^27^, our configuration does not require active finger contact with the textile electrode to initiate measurement, functioning akin to a cardiac patch for precise capture of cardiac electrical activity.

Concurrently, the IPG module employs a four-electrode configuration (Fig. 1a) to assess conductivity variations in the arm driven by blood flow dynamics, providing a robust non-invasive method for blood volume quantification. Typically, IPG measures impedance variations driven by pulsatile blood flow, where increased blood volume reduces impedance due to higher conductivity. A tetrapolar IPG configuration was employed to enhance measurement accuracy by separating current injection from voltage sensing, reducing contact impedance effects compared to two-electrode systems. Two outer electrodes (I + , I − ) inject a 12.5 kHz, 400 μA alternating current^28^, while two inner electrodes (V + , V − ) measure the resultant voltage gradient. These electrodes, integrated into the textile using silver-coated yarn (Fig. 1c), are arranged linearly around the upper arm with 2 cm spacing, aligning with the brachial artery’s longitudinal axis to optimize sensitivity to arterial blood volume changes. Based on this, the textile electrodes (Fig. 1c) facilitate simultaneous acquisition of dual-channel ECG and IPG signals, as shown in Fig. 1a, providing signal-level parameters such as dz/dt (first derivative of the measured IPG signal), pulse transit time (PTT), RR and QT interval, etc.

To be specific, we measure the cardiovascular metrics through exploring the parameters in circulation (detail derived in the Methods section), where these parameters including cardiac parameters (i.e., QTc, RRI and HR), vascular dynamic parameters (e.g., SV, SVR, BP, and PTT) extracted from the IPG and ECG signals (Fig. 1b). The cardiac parameters can be directed extracted from ECG signals while the vascular parameters should be determined by IPG or both signals. The PTT is the pulse generated from the heart to the measured site of the TAESS and can be determined by the ECGs’ R-peak to the peaks of IPG signals in each cardiac cycle. The SV^29^ can be determined directly from the IPG signals by Eq. (1).

1 $${SV}=\rho \,\cdot \,{L}^{2}/{Z}_{0}^{2}\,\cdot \,{dZ}/{{dt}}_{\max }\,\cdot \,{LVET}$$

where $\rho$ is the resistivity of blood (Ohms·cm). *L* is length between inner band electrodes (cm). Z_0_ is the base impedance corresponding to non-time varying tissues. ${dZ}/{{dt}}_{\max }$ is the magnitude of the largest impedance change during systole (Ohms/sec). ${LVET}$ is the systolic ejection time (seconds).

Furthermore, SVR can be determined by complex combination of the features from both electrical signals by Eq. (2). Later, we will validate its consistency with standard SVR measurement method^30^.

2 $${SVR}=\alpha \,\cdot \,{Z}_{c}\,\cdot \,{T}_{c}/{T}_{s}$$

where Zc is characteristic impedance, $\alpha$ is a BP-independent constant, and Ts and Tc are systolic duration and cardiac cycle^31,32^, details see Methods.

Therefore, the mean blood pressure (MBP) is directly calculated by the product of the HR, SVR, and SV as in Eq. (3) and systolic (SBP) and diastolic (DBP) pressures are derived through established MBP mapping relationships^33^.

3 $${MBP}={HR}\,\cdot \,{SV}\,\cdot \,{SVR}$$

Figure 1b illustrates a screening of the comprehensive cardiovascular heamodynamics based on the TAESS monitoring.

Additionally, by integration of advanced textile engineering into our dual-mode sensing system yields highly stretchable fabric electrodes woven with silver-based conductive yarns, delivering exceptional conductivity and mechanical durability. In the following, we will show that these electrodes surpass conventional commercial electrodes in signal fidelity and robustness. Later we will demonstrate the additional functional characteristics of this TAESS, including sweat permeability, stretchability, and thermal regulation (Fig. 1c). They exhibit outstanding repeatability and maintain stable acquisition of cardiac and vascular-related parameters—QTc, RRI, SV, HR, SVR and BP—under challenging real-world conditions, including humidity, motion, bending, and repeated reapplication. Consequently, the textile-based electrode configuration significantly outperforms traditional cuffless BP monitoring solutions, offering a scalable, low-cost platform suitable for daily use and enabling personalized, non-invasive cardiovascular monitoring in diverse settings (Fig. 1d).

### Fabrication and performance evaluation

**Fig. 2:**
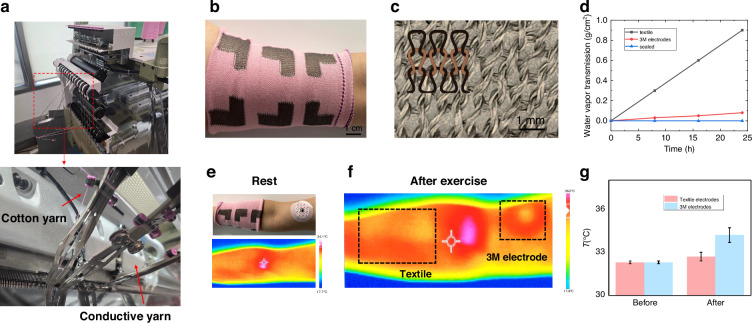
TAESS electrode design and characterization. **a** Automatic knitting machine. The conductive yarn is seamlessly knitted into the textile structure using conventional cotton thread. **b** TAESS electrodes worn on the arm. The gray patches are woven TAESS electrodes. **c** Microscopy image of conductive textiles and its structure illustration (inset). **d** Water vapor transmission rate of TAESS electrodes, 3 M electrodes, and sealed plastic film; The heat dissipation comparison between two type of electrode in the same arm at rest (**e**) and after exercise (**f**). **g** The magnitude of temperature difference before and after exercise (*n* = 6)

The textile electrodes were fabricated using programmable knitting technology (Fig. 2a) to form a six-electrode array designed for dual-mode integration^34^, consisting of two ECG electrodes and four IPG electrodes (Fig. 2b). The knitted structure incorporated interlaced conductive yarns arranged in interconnected loops, which offered the following advantages: (1) mechanical elasticity provided by the inherent deformability of the looped architecture, as illustrated in the microstructure inset of Fig. 2c; and (2) controlled free volume between yarns, enabling enhanced water vapor permeability while preserving electrical continuity.

To assess the permeability of the TAESS, we employed the water vapor transmission rate (WVTR) as a standardized metric, adhering to the ASTM E96 protocol. This evaluation is pivotal for ensuring cutaneous comfort and preventing moisture accumulation during extended wear, a critical consideration for wearable health monitoring systems^35^. The WVTRs of TAESS textiles, commercial 3 M electrodes, and sealed plastic were quantified as 37.5, 3.3, and 0 mg·cm^−^²·h^−^¹, respectively, as depicted in Fig. 2d. The TAESS’s exceptional permeability, attributed to its porous, woven architecture of silver-based conductive yarn, facilitates efficient moisture evaporation, markedly surpassing the occlusive properties of 3 M electrodes and non-permeable plastic. This superior permeability underscores the TAESS’s suitability for prolonged, non-irritating skin contact, enhancing its practical utility in continuous cardiovascular monitoring across clinical and home-based settings.

Subsequently, to elucidate the thermal performance of the TAESS, we investigated its heat dissipation characteristics, a critical factor for ensuring safety and comfort during prolonged electrode-skin contact, especially during exercise. Challenges such as heat generation and charge accumulation, exacerbated by sweating, pose significant risks for conventional electrode systems^10^. We conducted comparative thermal profiling using a high-resolution infrared imaging camera (Fluke Ti480P) to assess temperature distributions of TAESS and commercial 3 M electrodes before and after exercise (duration ~0.5 hours). The TAESS exhibited remarkable thermal stability, as evidenced by a consistent temperature profile (Fig. 2e), with snapshot measurements (Fig. 2f) confirming negligible heat buildup (<0.4 °C, averaged over multiple trials (*n* = 6), see Fig. 2g). No temperature correction or post-processing was applied, ensuring the values represent the TAESS’s intrinsic heat dissipation performance. This underscores its suitability for extended wear without risk of thermal-induced cutaneous irritation. In contrast, 3 M electrodes displayed pronounced heat accumulation, with a temperature differential of approximately 2 °C (Fig. 2g). This disparity is likely attributable to the dense, occlusive structure of conventional electrodes, whereas the TAESS’s woven, porous architecture, featuring silver-based conductive yarn, facilitates superior heat dissipation and moisture wicking, mitigating adverse skin effects and enhancing user safety.

In addition, we simulated the thermal state of a human wearing TAESS in Fig. S2 to further evaluate its thermal regulation performance. We conducted controlled experiments using a thermally regulated platform (40°C) and 3-mm-thick PDMS skin simulation material. Under simulated heating conditions, TAESS maintained a stable skin proximal temperature of 37.0 °C, while the temperature of the commercial 3 M electrode was 38.5 °C (Fig. S2), indicating that TAESS exhibits superior thermal regulation performance under simulated heating conditions. To assess environmental adaptability, we tested TAESS under post-exercise conditions at room temperatures ranging from 20 to 28 °C (controlled by indoor air conditioning, see Fig. S3). The results showed that thermal regulation accuracy remained within the range of 0.3–0.6 °C across all scenarios. These findings indicate that TAESS can achieve thermal regulation functionality in real-world scenarios.

### Robustness of multi-modal signal acquisition

**Fig. 3:**
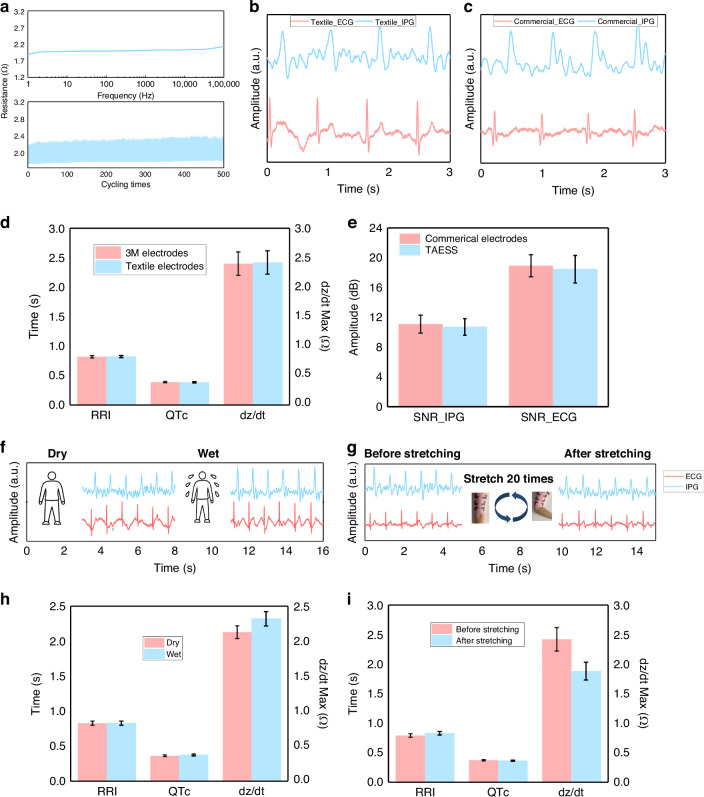
Wearability characteristics of fabric electrodes in multi-scenario comparative tests. **a** Electrical characteristics of the TAESS electrodes and resistance variations of TAESS electrodes under different strains. **b**, **c** The ECG and IPG measurement by textile electrodes of TAESS and commercial electrodes. **d** The cardiovascular metrics (e.g., RRI, QTc, and dz/dt) from textile electrodes of TAESS. **e** SNR comparisons of ECG and IPG signals from TAESS and commercial system. **f** The permeability and conductivity evaluation of the TAESS. **g** The stretchability evaluation of the TAESS. **h** The cardiovascular metrics comparison between the dry and wet states of the TAESS and (**i**) before stretching and after stretching (20 times)

We now turn to investigate the TAESS in multi-modal signal acquisition across diverse physiological scenarios, including dry and hydrated conditions as well as mechanical stretching. The electrical characterization was conducted across a frequency spectrum of 1 Hz to 100 kHz, as depicted in Fig. 3a, strategically encompassing: (1) high-frequency (10–100 kHz) impedance plethysmography (IPG) measurements using four electrodes to optimize tissue penetration depth, and (2) low-frequency (1–100 Hz) electrocardiography (ECG) recordings via two electrodes to capture cardiac spectral components. To refine electrode architecture, we tailored knitting loop intervals (0.8–1.8 mm), as shown in Fig. S4, modulating textile density while maintaining conductivity, with minimal resistance increases observed across larger intervals, highlighting structural resilience (Fig. S4). The TAESS exerts an estimated skin pressure of approximately 0.5–1.5 kPa, calculated based on the elastic modulus of its stretchable textile architecture (Young’s modulus ~0.1 MPa) and its design to fit arm circumferences of 20–30 cm. This pressure range, comparable to medical compression garments^36^, ensures intimate electrode-skin contact for robust ECG and IPG signal acquisition while remaining below the threshold for discomfort or restricted blood flow (typically >6 kPa)^37^. Fig. S5 demonstrates full electrical recovery post-45% tensile strain (in wale direction and 30% strain in course direction), while 500 stretch–release cycles (Fig. 3a) confirm mechanical durability, with transient resistance fluctuations during deformation—attributable to conductive pathway realignment—reverting to baseline, underscoring exceptional reusability. The porous textile structure further enhances permeability, surpassing commercial 3 M electrodes.

We subsequently evaluated the TAESS’s performance under real-world wear conditions, assessing its capacity to maintain signal fidelity and extract precise cardiovascular metrics in diverse scenarios. Comparative experiments benchmarked TAESS against the Biopac system, with TAESS and 3 M electrodes simultaneously acquiring ECG and IPG signals (Fig. 3b, c) at identical arm positions. Statistical analysis (paired t-tests, *p* > 0.05) revealed no significant differences in ECG-derived features (RRI, QTc) or IPG parameters, affirming data consistency (Fig. 3d). The quantitative evaluations of signal-to-noise ratio (SNR) in Fig. 3e. show the TAESS’s ECG-SNR at 19 dB (vs. 20 dB for Biopac) and IPG-SNR at 11.8 dB (vs. 12 dB for Biopac), confirming comparable performance. To probe performance under hydration, ECG and IPG signals were recorded in dry and hydrated states, revealing distinct arterial impedance and cardiac rhythm profiles (Fig. 3f, h). Stretchability was validated through controlled extension trials, with cardiovascular metrics remaining numerically stable pre- and post-stretching (Fig. 3g, i), despite minor reductions in peak dZ/dt, potentially due to transient vascular flow alterations. Besides, despite the complex and non-uniform deformation of the fabric sleeve caused by muscle expansion during physical activity, the TAESS electrode demonstrates a superior SNR compared to the 3 M electrode in ECG and IPG signals, see Fig. S6. In addition, we incorporated experimental procedures simulating dynamic conditions, including walking and arm swinging, to validate the performance of TAESS under these circumstances, see Fig. S8. These rigorous validations establish the TAESS as a multifunctional platform for continuous, multi-parameter cardiovascular monitoring, laying a robust foundation for advanced haemodynamic assessments.

### Alignment-free blood volume detection

**Fig. 4:**
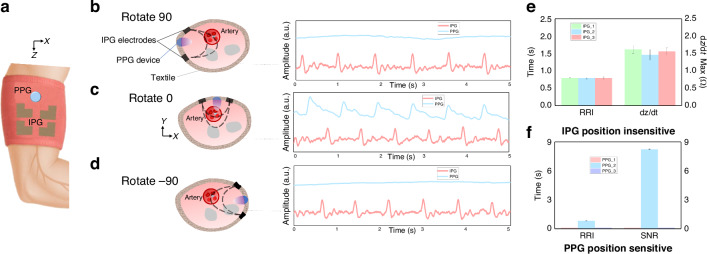
Alignment-free of TAESS in wearable haemodynamic parameter measurement. **a** Setup of sensor in the arm. Rotating the IPG electrode radially by (**b**) 90 degrees, (**c**) 0 degrees, and (**d**) −90 degrees, while comparing the impact of the PPG device on blood flow signal acquisition. **e** Quantified RRI and dz/dt under rotation. **f** RRI and signal-to-noise ratio of the PPG under sensor rotation

We further validate the positional robustness of the TAESS of the blood volume measurement. We employed an isotropic rotating sleeve configuration (Fig. 4a) integrated with impedance plethysmography (IPG) and conducted comparative assessments against high-sampling rate (1000 Hz) commercial photoplethysmography (PPG) sensors. Unlike commercial PPG sensor, which are constrained by their dependence on precise vascular alignment^38,39^ due to low skin penetration depth of optical method, TAESS-derived blood flow measurements exhibit exceptional rotational invariance, a pivotal attribute for alignment-free cardiovascular monitoring. Controlled experiments, depicted in Fig. 4b–d, evaluated IPG performance at rotational angles of 0°, +90°, and −90°, revealing consistently discernible blood flow waveforms with minimal deviation in derived physiological parameters, such as RRI and dz/dt values in Fig. 4e. In contrast, PPG sensors, limited to superficial vascular interrogation (Fig. 4b–d), produced negligible signals upon even slight positional displacement from the vasculature, as illustrated in Fig. 4f.

The TAESS’s resilience to positional variability was further substantiated through two critical metrics: (1) Axial displacement analysis of IPG signals demonstrated stable peak intensities across positional shifts (~2 cm each step), ensuring reliable signal capture irrespective of sleeve orientation, Fig. S7. (2) Radial rotation assessments confirmed negligible variance in peak intensity and dynamic waveform properties, underscoring the system’s robustness. This positional independence, enabled by the TAESS’s flexible, silver-based conductive yarn electrodes, facilitates seamless integration into daily wear, mitigating the alignment challenges inherent in conventional wearables. Besides, we also conducted the controlled tests involving common daily activities such as walking and arm swinging, simulating everyday scenarios to evaluate the motion artifacts. Compared to a Biopac MP160 system with 3 M medical-grade electrodes, the TAESS exhibited robust signal fidelity, with transient fluctuations during motion recovering within 2–3 seconds post-movement (Fig. S8). The lightweight wire connections of the TAESS system greatly reduce the impact of wire pulling during movement compared to the hard wire connections of the Biopac system, allowing the ECG and IPG SNR to remain stable after movement recovery (Figs. S8 & S9).

### Comprehensive multi-parameter cardiovascular monitoring

In addition to the TAESS electrodes, which utilize the electrical conductivity of blood (a property independent of vascular location) to achieve unrestricted signal acquisition, the sleeve-based sensor also provides a unique SVR assessment, facilitating accurate measurement of blood pressure and hemodynamic parameters.

**Fig. 5:**
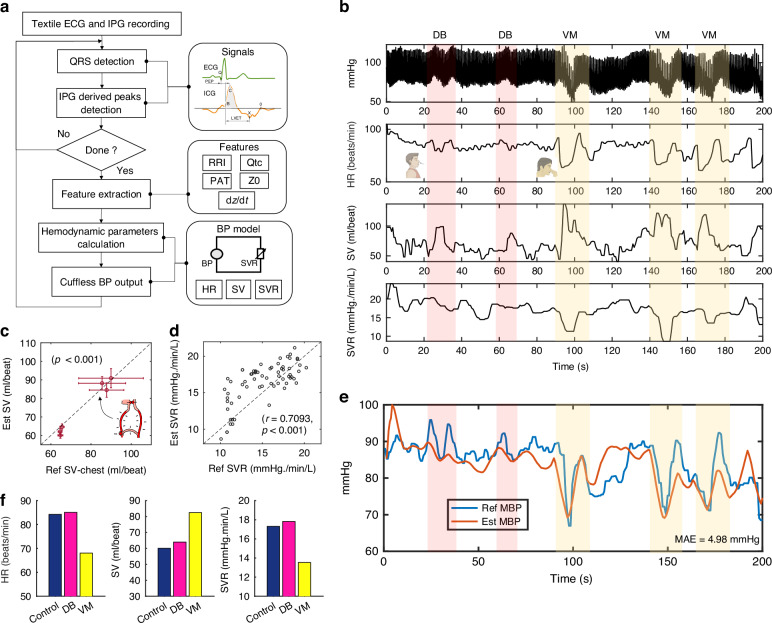
Cardiovascular multi-parameter tracking by TAESS system. **a** The flowchart of the TAESS system in BP measurement. **b** An example of the TAESS system in cardiovascular heamodynamic measurement during random respiratory interference from a participant. **c** The relation of the TAESS-based SV (Est SV) and the gold standard SV-chest (Ref SV-chest); the higher variation of the SV can be generated during the respiratory interference as shown in (**b**). **d** The relation of the TAESS-based SVR (Est SVR) and the gold standard SVR (Ref SVR) (r = 0.7093, *p* <0.001). **e** The prediction of the MBP based on our TAESS system when compare to the reference MBP from the commercial NIBP100D device; (**f**) the comparison of the core parameters in HR, SV, and SVR (Average within ~10 seconds duration) across resting (control), deep-breathing (DB), and valsalva maneuver (VM)

To validate this functionality, we induced SVR changes by introducing different breathing patterns (baseline resting, deep breathing, and breath-holding or valsalva maneuver) to assess its ability to capture changes under different blood pressure conditions. Using sleeve-based IPG electrode technology, we achieved continuous and synchronous tracking of composite haemodynamic parameters. Figure 5a details the complete calculation and extraction process of haemodynamic indicators. By extracting features from the signals and substituting them into Eq. (1-3), we derived synchronized beat-by-beat BP, SV, and SVR values. Figure 5b illustrates a testing protocol involving multiple breathing patterns, where a participant wore TAESS and randomly performed different breathing patterns, including resting, deep-breathing (DB), and valsalva maneuver (VM). Figure 5c presents the correlation between sleeve-based blood volume and whole-body cavity blood volume^28^. The x-axis represents the standard measured stroke volume, while the y-axis displays the estimated whole-body stroke volume derived from sleeve measurements. Despite significant blood flow disturbances induced by respiration, the concordance between estimated and measured SV is apparent. In addition, SVR was corrected using two sets of BP values to eliminate the influence of physiological BP-independent parameters, such as blood density and conductivity. Figure 5d demonstrates that the estimated SVR from Equation (3) aligns with measured SVR that calculated by MBP/CO from the commercial Biopac system. The haemodynamic parameters corresponding to different breathing patterns are presented in Fig. 5e. The results obtained from the blood pressure calculation model are compared with the measured results in Fig. 5f, with an mean absolute error (MAE) within 5 mmHg. Moreover, we recruited 10 participants and conduct a random respiratory and re-wearing interference experiments. The Bland-Altman plots shown in the Fig. S10, exhibiting a MAE of 4.1399 mmHg when cover a large range of MBP in [60,100] mmHg. Finally, we systematically compared our method with the classical PWV method^40^ and the zero-baseline method^41^. Our method achieved an unbiased root-mean-squared-error (RMSE, *n* = 3583) of 7.05 mmHg and 5.93 mmHg of SBP and DBP, whereas the PWV method had a RMSE of 10.13 mmHg and 8.59 mmHg of SBP and DBP, the zero-baseline benchmark had 12.24 mmHg and 8.64 mmHg of SBP and DBP, respectively. The position-sensitive PWV method may produce the greatest errors due to changes in PTT caused by respiratory and re-wearing interference. In addition, given that current physiological methods rely solely on the relationship between a single parameter and BP, this simplified approach limits their tracking capability. In contrast, our TAESS system directly captures core feature quantities, enabling it to maintain high accuracy. Finally, to compare the performance of the TAESS system with that of 3 M commercial reference systems, we also show the good alignment trajectories of MBP, HR, and SV for both systems over the entire 400-second time window, see Fig. S11. The average MAE of 4.03 mmHg for MBP, with a standard deviation of 0.67 mmHg (Fig. S12), confirms consistent performance across subjects. These findings offer novel solutions for developing cardiovascular haemodynamic measurement methods within a smart sleeve.

## Discussion

This study introduces a textile-based alignment-free electrophysiological sensing sleeve (TAESS) that combines low-cost, automated knitting processes with advanced material properties to create a scalable and wearable platform for continuous cardiovascular monitoring. The fabric electrodes, woven from silver-based conductive yarn, offer superior sweat permeability, thermal regulation, and stretchability, ensuring excellent comfort and wearability for daily use. Furthermore, the alignment-free feature is achieved by acquiring electrophysiological signals at the upper arm, unlike PPG or ultrasound-based systems that require precise alignment with arteries. This relaxed alignment requirement, combined with the exceptional wearability of the fabric, enables seamless integration into daily wear while addressing common challenges in traditional systems, such as discomfort, alignment sensitivity, and motion artifacts.

Notably, the impedance plethysmography (IPG) module of TAESS monitors local blood volume changes in the brachial artery. Since the anatomical structures of the left and right upper limbs are similar, there is no significant difference between them. However, for electrocardiogram (ECG) measurements, since the left arm is closer to the heart (~ 24 cm vs. ~32 cm for the right arm), and cardiac potential weakens inversely with increasing distance^42^, this geometric advantage facilitates the detection of high-quality ECG signals (Fig. S13), which is critical for accurate monitoring of heart rate and rhythm. It is also worth noting that this study is related to common physiological states, such as deep breathing, Valsalva maneuvers, walking, and arm swinging, etc. However, extreme physiological states, such as rapid blood pressure fluctuations, may necessitate higher sampling rates. Consequently, future investigations should employ elevated sampling rates (e.g., 2 kHz) to assess performance under these conditions. Although our current study was limited to single-arm electrocardiogram (ECG) and impedance cardiography (IPG) assessments in a cohort of healthy individuals to derive essential hemodynamic parameters, future research will expand the scope of TAESS to diagnostic applications, including arrhythmia detection^43^ via single-arm electrocardiograms.

Beyond device manufacturing and design, the key enabling component of this work lies in its physiological modeling, which leverages the interplay between IPG and ECG signals to extract comprehensive cardiovascular parameters. By combining these dual-modal signals, the system enables the precise BP measurement of blood pressure and introduces a novel approach to estimating SVR—a parameter challenging to measure using traditional methods^11,44,45^. Our results demonstrate that the TAESS maintains BP tracking accuracy even after re-wearing or under respiratory interference. In doing so, the system supports comprehensive cardiovascular assessment by providing a feature array of BP, CO, SV, HR, and SVR. These advancements position the TAESS as a transformative solution for continuous, alignment-free cardiovascular monitoring, enabling personalized healthcare in daily life.

## Methods

### Fabrication process

The conductive yarn used in the TAESS is a commercially available silver-coated cotton yarn (350D, Shengxin, China, https://www.zgxjjypt.com/com/shengxin8899/). It consists of a cotton core with a silver coating (> 18% silver content), offering a balance of conductivity (resistance: 1.4–1.6 Ω/cm), flexibility, and softness suitable for machine sewing. The yarn has a diameter of 0.172–0.181 mm, an elongation of 10%, and a breaking strength of 1.28 kgf at 30 cm, ensuring durability against mechanical stress during stretching (up to 45% strain in wale direction and 30% strain in course direction, Fig. S5). This yarn is seamlessly knitted into the TAESS’s porous textile structure using conventional cotton thread. In details of the fabrication process, the yarn goes through the tension equipment and the side tension equipment. To prevent the bottom edge of the garment from unraveling and to allow for pulling, the fabric must first be knit into a row of starting loops using a rubber band. The fabric comb will hook the rubber band and pull the fabric downwards. The fabric comb releases the fabric, and the pulling roller will pull the fabric downward. The carriage controls the motion of the needle and knitting speed. After the rubber band in the first row, the fabric requires around 60 rows for the support section. The support section makes sure the fabric comb can pull the fabric downwards. After the support section, the machine will knit the fabric. The TAESS dimensions are based on universal sizes that fit upper arm length and circumference. Each TAESS electrode patch was engineered with a surface area of approximately 6 cm². The parallel configuration of electrode patches was implemented to ensure stable IPG and ECG measurement.

### Characterization

An impedance analyzer (IM7581, HIOKI) was used to measure the impedance across a broad frequency range from 1 Hz to 100 kHz. To test stretchability and mechanical durability, a platform to test the resistance variations under different strains was set up, including a fixed stage, a uniaxial moving stage, a displacement sensor (UM12-1172261, Sick), and a sourcemeter (model 2612 A, Keithley). During the test, one end of the textile electrode was fixed on the fixed stage, and the other end was fixed on the moving stage. The sourcemeter measured the current flowing through the sensor under a constant voltage. The water vapor transmission rate was evaluated by measuring the weight of water in a bottle, when the opening is covered by the textiles, 3 M electrodes and sealed plastic film respectively. The detailed process was as follows: three bottles were filled with 20 ml of distilled water with an open diameter of 15 mm, then three materials were attached to the opening of the bottle using adhesive tapes. The bottles were then placed in a chamber with a temperature of 20 °C. The mass of bottles was measured every 4 h. The water vapor transmission rate was calculated based on mass change. The experimental arrangement for temperature field testing comprises the application of a thermography device (Fluke Ti480P). To commence, textile electrodes and 3 M electrodes were affixed to the skin. Subsequently, conduct thermal imaging using the thermography device before and after exercise respectively.

Hydration testing was conducted by applying water to the TAESS’s electrode area via controlled spray coating until full saturation, simulating extreme sweating conditions exceeding typical human sweat rates (0.5–1 L/h from low to moderate exercise)^46^. Approximately 5 mL of water was uniformly applied to the electrode region (covering six electrodes), ensuring complete wetting of the textile without pooling. These tests were performed in both dry and fully hydrated states.

### Dual-mode electrical system data acquisition

A Biopac MP150 system (BIOPAC system inc. California, USA) with ECG100C, EBI100C, and Cnap-NIBP 100D modules for the data collection. All ECG, IPG and BP were simultaneously recorded at different physiological states (resting vs. deep breathing). The textile impedance electrodes were worn at the arm side, and it was plugged in the EBI100C module to measure impedance (at a 12.5 kHz measurement frequency and a precision high-frequency current source, which injects a very small (400 µA) current through the measurement tissue volume defined by the placement of a set of current source electrodes). A separate set of monitoring electrodes then measures the voltage developed across the tissue volume. Because the current is constant, the voltage measured is proportional to the characteristics of the biological impedance of the tissue volume. On the other hand, we use ECG100C module to measure the heart’s electrical activity through skin electrodes on the arm. We recruited 10 young subjects for data collection (details in Table S1) to validate the function of the textile-based dual-mode electrical BP measurement system. The subjects were in a rest state before the measurement; during the measurement, the subjects were asked to take several deep breaths or Valsalva maneuver at random time points or re-wearing during the measurements. All data was recorded with a sampling frequency of 1000 Hz. This experiment was approved by the Joint Chinese University of Hong Kong-New Territories East Cluster Clinical Research Ethics Committee. The consent forms were completed by the subjects prior to the experiments.

### Computational model

To enable comprehensive cardiovascular haemodynamics measurement, we first describe the cardiac parameters extracted from ECG signals, including QTc, RRI, and HR. The QTc=QT/sqrt(RRI), and RRI is the RR-interval of ECG signal. HR is defined as 60/RRI (beat-per-minute). Then we describe SV, which is determined by the IPG measurement and expressed as in Eq. (1). Z_0_ is the base impedance corresponding to non-time varying tissues, such as muscle, bone and fat. It is measured when the pulsatile volume is minimal. Furthermore, we derived SVR as in Eq. (2), i.e., ${SVR}={Z}_{c}\,\cdot \,{\kappa }^{-1}$, where $\kappa$ is the parameter of the systemic reflection coefficient (SRC)^31,32^, i.e., $\kappa ={Z}_{c}/{SVR}$, and can be further expressed as $\kappa =\alpha \,\cdot \,{T}_{s}/2{T}_{c}$, and $\alpha =({P}_{i}-{DBP})/({MBP}-{P}_{o})$ is regarded as a BP-independent value that can be determined with a pair of BP values as in the initialization. Zc is the characteristic impedance. ${T}_{s}$ is the systolic time interval and can be determined by QT from the ECG signal; similarly, ${T}_{c}$ is the RR-interval from ECG signal. ${Z}_{c}=\eta \,\cdot \,{PWV}/L\,\cdot \,Z\,\cdot \,{\kappa }^{-1}$. where $\eta$ is the ratio of blood density and conductivity. Z is the impedance value from the IPG signal. L is the measured length of the pressure pulse transmission. The units for SVR are expressed as pressure (mmHg) divided by cardiac output (L/min), i.e., mmHg⋅min⋅L^−^^1^. Finally, we employ a decoupled haemodynamic model based on the classical Ohm’s law^30^ of fluid equation in Equation (3) to derive BP. Notably, to ensure robust signal integrity and precise extraction of cardiovascular metrics, all output cardiovascular data from the TAESS were processed using a 5-point moving average and median filter.

### Statistical analysis

Signal parameters were extracted and calculated using MATLAB R2022a. Various statistical analysis metrics were calculated, including B-A plot and correlation plots were produced using MATLAB. Simple correlation analysis was performed using the Pearson correlation method, and methodological consistency was tested using the pair two-sample *t*-test. Statistical significance was indicated by *p* <0.05 (*), <0.01 (**), and <0.001 (***). The mean absolute error (MAE) and root-mean-squared-errors (RMSE) were used to evaluate the accuracy of TAESS for BP estimation.

## Supplementary information

Supplementary Information
