# Supplementary material for: A textile-based alignment-free electrophysiological sensing sleeve for comprehensive cardiovascular monitoring
Source: Microsyst Nanoeng. 2025 Nov 26;11:228. doi: 10.1038/s41378-025-01088-x (PMC12647701; doi:10.1038/s41378-025-01088-x)
Supplement: Supplementary file 1 — Supplementary Information [file 41378_2025_1088_MOESM1_ESM.docx]

**A Textile-Based Alignment-Free Electrophysiological Sensing Sleeve for Comprehensive Cardiovascular Monitoring**

Shirong Qiu^1†^, Yihao Li^1†^, Chenkai Dai^5^, Shun Wu^1^, Xiangjia Chen^2,5^, Nan Ji^4^, Guoxin Fang^2,5^, Yeung Yam^2,5^, Charlie CL Wang^3^, and Ni Zhao^1*^

*Correspondence: nzhao@ee.cuhk.edu.hk.

^1^ Department of Electronic Engineering, The Chinese University of Hong Kong, Hong Kong SAR, China

^2^ Department of Mechanical and Automation Engineering, The Chinese University of Hong Kong, Hong Kong SAR, China

^3^ Department of Mechanical and Aerospace Engineering, The University of Manchester, Manchester, M20 4BX, UK

^4^ United Sensing and MediTech Limited, Hong Kong SAR, China

^5^ Centre for Perceptual and Interactive Intelligence (CPII) Limited, Hong Kong SAR, China

^†^ These authors contributed equally.

**Supplementary Figure S1:**

*Figure S1. Biophysical rationale for upper arm ECG acquisition based on the cardiac vector projection during the heart’s electrical activity.*

**Supplementary Figure S2:**

*Figure S2. The thermal regulation evaluation of both 3M and TAESS electrodes on the PDMS skin under 40°C of simulated fever conditions. The higher thermal (1.5°C) in the position of 3M when compared to the TAESS patch.*

**Supplementary Figure S3:**


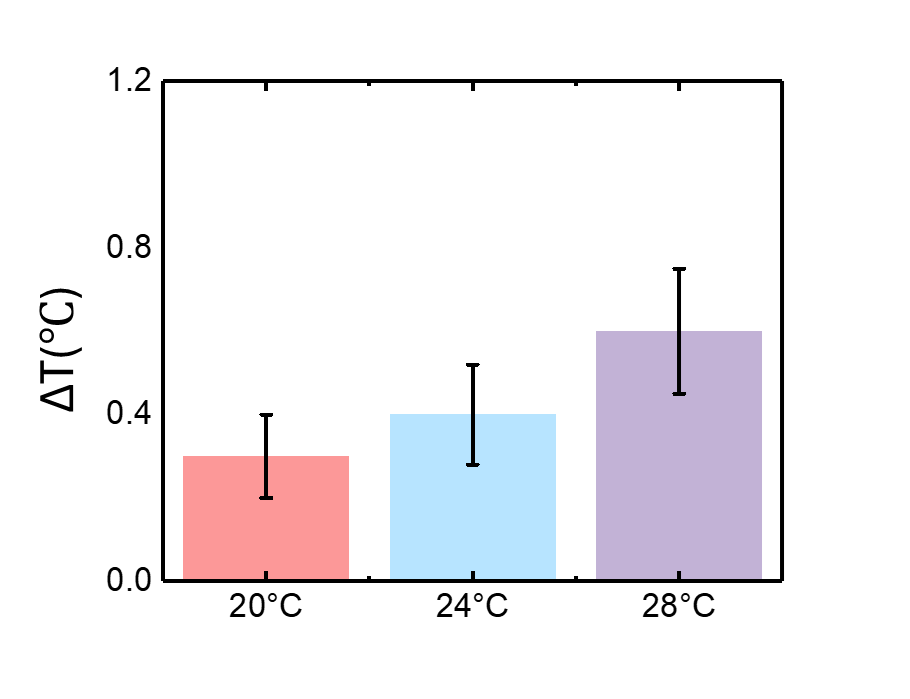


*Figure S3. The temperature control experiment for evaluate the environmental robustness of TAESS electrodes.*

**Supplementary Figure S4:**

*Figure S4. I-V curve of textiles made at different hole intervals including 0.8mm, 1.3mm and 1.8mm.*

**Supplementary Figure S5:**

*Figure S5. Resistance variations of TAESS electrodes under different strains.*

**Supplementary Figure S6:**


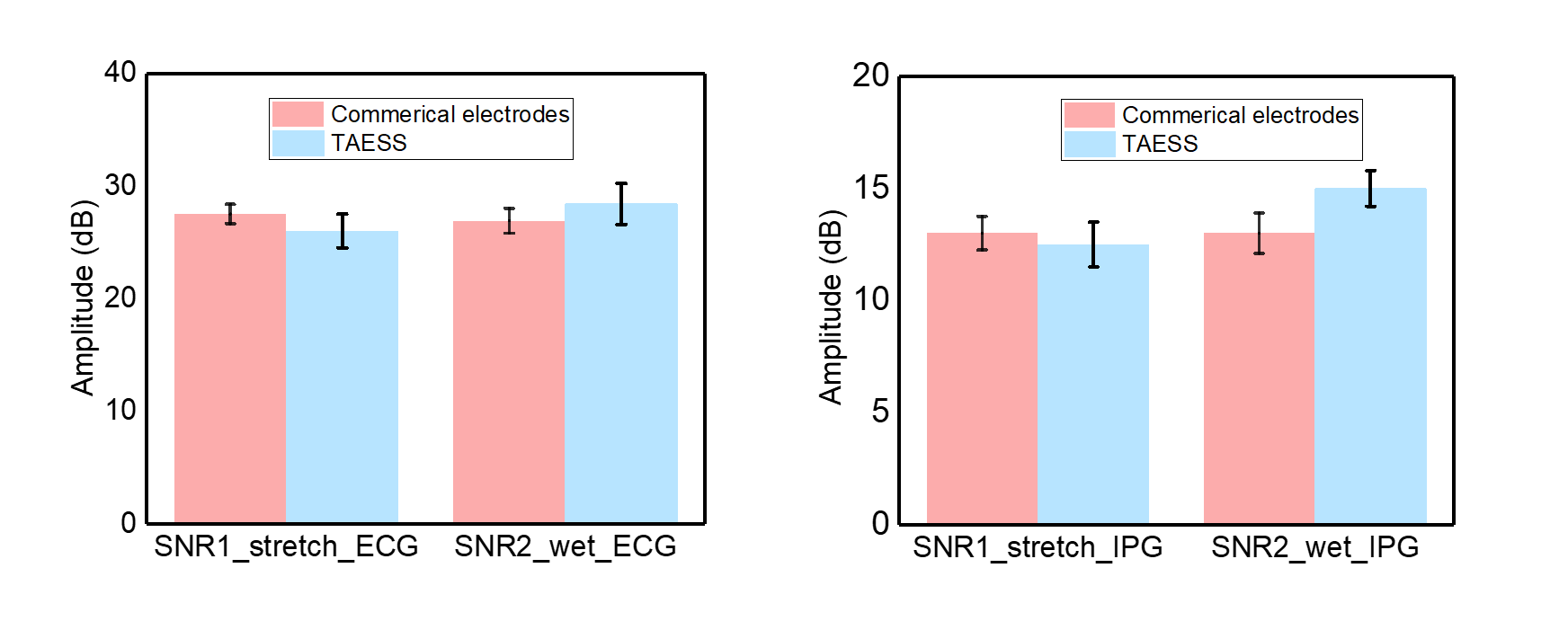


*Figure S6. SNR1_strech is the SNR before and after stretching. SNR2_wet is the SNR in dry and hydrated states.*

**Supplementary Figure S7:**


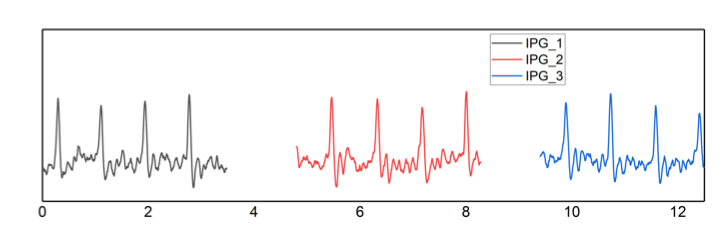


*Figure S7. Different IPG signals along axial displacement.*

**Supplementary Figure S8:**

*Figure S8. Comparisons of the ECG and IPG signals measurement of TAESS and 3-M based commercial reference system in different real-world scenarios, including (a-b) the resting monitoring; (c-d) arm swinging monitoring; and (e-f) walking and arm swinging.*

**Supplementary Figure S9:**


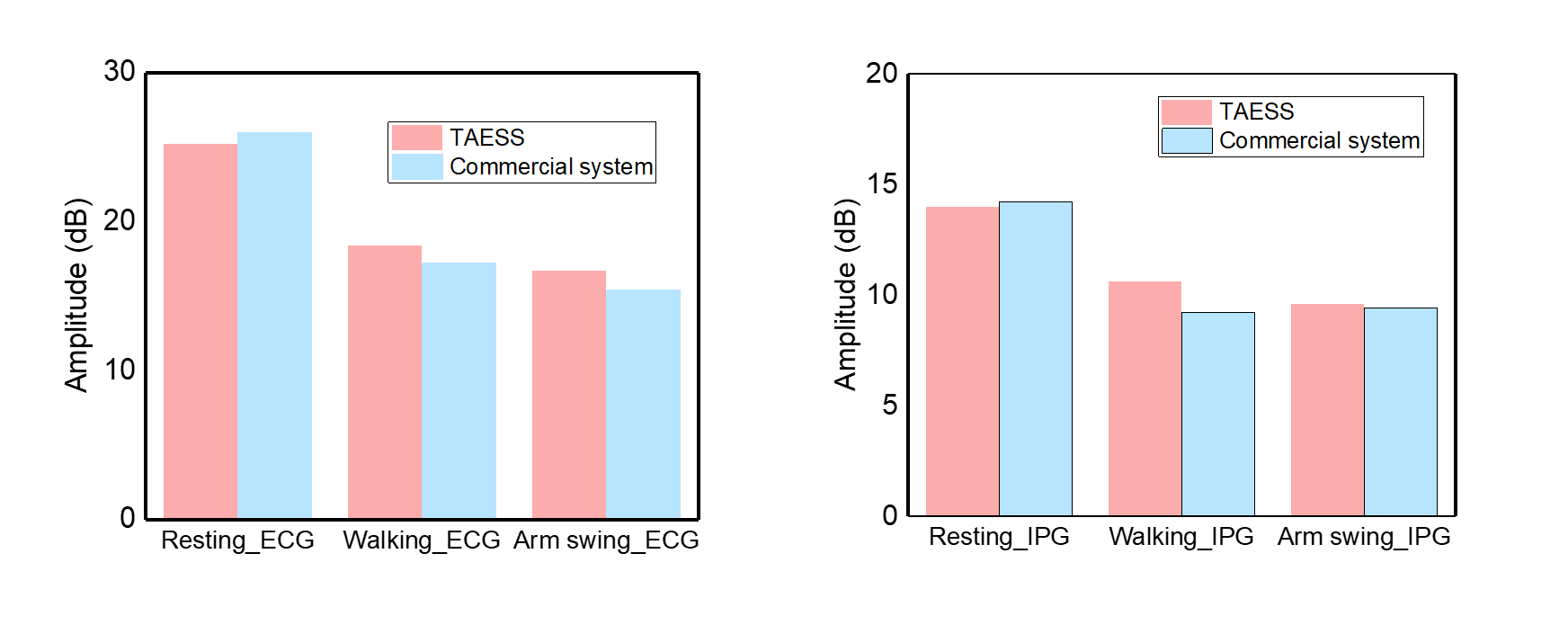


*Figure S9. Comparisons of the SNRs of ECG (left panel) and IPG (right panel) signals measurement of TAESS and medical-grade 3M based reference system in different real-world scenarios.*

**Supplementary Figure S10:**

*Figure S4. Bland-Altman plots of MBP of TAESS-based BP method in the 10 recruited subjects (N=3583 beats) under random respiratory and re-wearing interference.*

**Supplementary Figure S11:**

*Figure S11. Comparison between commercial reference system and the TAESS system in the vital cardiovascular parameters extraction (e.g., MBP, SV, and HR).*

**Supplementary Figure S12:**

*Figure S12. The MAE distribution across 10 participants.*

**Supplementary Figure S13:**

*Figure S13. The comparison of the ECG measurement on the left and right upper arms.*

*Table S1. Demographic characteristics of the study population (n=10).*

| Age (yrs) | Gender (male/female) | BMI | Height (cm) | Weight (kg) |
| --- | --- | --- | --- | --- |
| 28$\pm$3.23 | 8/2 | 21.39$\pm$1.44 | 171.2$\pm$5.96 | 62.7$\pm$5.79 |
